# Coronavirus-Infected International Business Transactions: A Preliminary Diagnosis

**DOI:** 10.1017/err.2020.30

**Published:** 2020-04-07

**Authors:** Marco TORSELLO, Matteo M. WINKLER

**Affiliations:** * University of Verona, Verona, Italy; ** HEC Paris, Jouy-en-Josas, France; email: winkler@hec.fr

## I. Background

Since the very start of 2020, international business actors have become increasingly concerned with the “viral exceptionalism”^1^ caused by the various governmental pandemic-mitigation restrictions (PMRs) enacted in response to COVID-19.^2^

The governmental action was deemed necessary as a consequence of coronavirus’ distinctive epidemiological characteristics. First, by replicating in large quantity during the prodrome period, the virus spreads via silent and unconscious carriers, who remain asymptomatic despite the infection.^3^ Second, the virus possesses both an extremely efficient human transmission capacity and a long asymptomatic incubation period (1–14 days, compared to 2–7 days of SARS), two factors that, combined, make it difficult to detect and quickly circumscribe the infected population within a limited geographical range.^4^ Third, a significant portion of infected individuals require immediate access to intensive care units, thus creating a burden for national public health systems, which are ill-equipped to face the resulting hospitalisation surge.^5^ Hence the need to urgently “flatten the curve” of the pandemic growth.^6^

In several countries, PMRs took the form of strict nationwide lockdowns (travel bans, prohibition of gatherings and closure of education facilities) and the suspension of all non-essential trade (anything but food, medicine, banking, financial and insurance services). Based on their systemic effects, such measures are extremely pervasive, as their impact on human rights is ubiquitous,^7^ although (presented as) temporary, as they will presumably remain in force until the pandemic is sufficiently mitigated. Most likely, compliance with such measures will result in a dramatic liquidity crisis followed by an economic recession. These economic considerations may have played a role in inducing some countries to adopt less stringent measures than a generalised lockdown and the discontinuance of most business and industrial activities.^8^

In any event, in all jurisdictions the issue arises as to the impact of COVID-19 on the ability of the parties to duly perform their contractual obligations. This circumstance is not confined to domestic settings, but extends to international business transactions, where the risks arising both from pandemics and from the subsequent PMRs are typically regulated through appropriate *force majeure* clauses.^9^ However, unlike the corresponding national provisions regarding *force majeure*, which tend to exclude the party’s liability for failure to perform, the above-mentioned clauses regulate the effects of *force majeure* events in several different fashions by offering the parties ways to preserve their contractual relationships.^10^

This article offers a threefold analysis of *force majeure* clauses in light of PMRs. First, it proposes an overview of *force majeure* clauses and their effects. Second, it assesses the possible use of such clauses in relation to PMRs. Finally, it examines the possible extraterritorial reach of such PMRs.

## II. COVID-19, pandemic-mitigation restrictions and *force majeure* clauses

### 1. How COVID-19 affects international contracts

While still in the midst of the pandemic, it is already possible to identify some critical impediments to contractual performance in international business transactions caused by COVID-19.

In some cases, COVID-19 may have a *direct* impact on performance, as in the event of the contractor (or the contractor’s employees) being infected with the virus, of a shortage of components being supplied from an area severely affected by the pandemic, of carriers refusing to travel to a certain area due to the outbreak of a cluster of cases or of a loss of interest by potential customers for an event (such as a show or a sporting event) where the risk of infection is high due to the uncontrolled gathering of people. Unlike cases of natural disasters, however, an epidemic – even when it reaches the span of a pandemic – could be regarded as not making performance per se absolutely impossible. In fact, the contractor could hire new employees, the manufacturer could purchase components from alternative sources of supply, the party required to arrange for transport could seek another carrier or choose another route and certainly a show and a sporting event are possible even behind closed doors.

Whereas the concrete (im)possibility of performance caused by COVID-19 may be questionable, this uncertainty is conveniently reduced when public authorities intervene by prohibiting certain activities, thus offering the obligor the option of invoking impossibility of performance on the basis not of the pandemic itself, but of the act of the authority (*factum principis*). If the government imposes quarantine on employees, prohibits imports from a certain area, closes frontiers or prohibits shows and sporting events, then no question arises as to the *legal* impossibility of performance, which lasts until the government decides to lift the ban or prohibition.

The impact of COVID-19, however, seems to go far beyond the direct effect described above. The question therefore arises as to whether a generalised change of circumstances, which may be deemed to be the *indirect* long-term consequence of COVID-19 and to produce consequences such as liquidity crises, price fluctuations and generalised supply difficulties due to the increased health risks, qualifies for an exemption from contract performance. Under these circumstances, businesses are increasingly wondering what their options are in the face of COVID-19 or of its indirect, large-scale economic consequences.

The correct answer to this question is likely to be that in most cases the *force majeure* clauses contained in international contracts cannot be stretched so as also to cover the said changes of circumstances, although it is possible that an exemption may be available under a different clause in the contract, if present, namely the *hardship* clause, which is, however, much less frequently included in international contracts.^11^

### 2. International contracts’ resilience to the COVID-19 pandemic

*Force majeure* clauses identify exceptional circumstances upon the occurrence of which the obligor is exempted from liability for failure to perform. Whether an event constitutes *force majeure* under a contract involves a highly fact-specific analysis. In most cases, a *force majeure* event must be unforeseeable and beyond the reasonable control of the obligor; moreover, the obligor must not be in the position to avoid or overcome the event or its consequences.

Under certain contracts, events that constitute *force majeure* are specifically listed and such lists may include epidemics, pandemics or other terms relating to severe widespread diseases, which clearly cover COVID-19. If the list is meant to be exhaustive, the absence of an express reference deprives the aggrieved party of the remedy. Several contracts, however, merely contain a general catch-all phrase referring to the requirements of *force majeure* mentioned above (or contain a merely illustrative list), thus leaving the question open as to whether the COVID-19 pandemic qualifies as a *force majeure* event under that specific contract.^12^

Even if COVID-19 falls within the contractual notion of *force majeure*, other requirements must be met, namely a direct causal link between the pandemic and the non-performance (which must be “due to” the pandemic)^13^ and the unavoidability of the effects of COVID-19 (or of subsequent PMRs), whereas it is debated whether the *force majeure* event must be unforeseeable.^14^ In some of the few comparable circumstances (ie upon the occurrence of other epidemics), courts have been extremely reluctant to grant the remedy.^15^

If all of these requirements are fulfilled and if the party affected by the impediment invokes *force majeure* in compliance with other contractual requirements (form, time limit), then the clause provides for a remedial scheme, which in most cases is based on the interplay of suspension of performance, exemption from liability for non-performance and termination of contract.

In particular, the party successfully invoking *force majeure* may *suspend* performance for the duration of the impediment, provided that the other party may also react by suspending its counter-performance. However, if the suspension of performance or the temporal extension thereof deprives either party of what it was reasonably entitled to expect under the contract, either party may *terminate* the contract.^16^ In any event, whatever the remedy may be between suspension and termination, the non-performing party is relieved from liability for damages, including liquidated damages for delay in performance and other contractual penalties.

In a smaller number of cases, the contractual remedial scheme may contain alternative solutions – more commonly to be found in *hardship* clauses – aimed at preserving the contract, namely a duty to *renegotiate* the terms of the transaction (which in itself does not imply a duty to reach agreement on new terms) or the endowment of a third party or a court with the power to *adapt* the contract terms to the changed circumstances.

Not only do these alternative remedies imply that the *force majeure* clause in question also covers situations where performance is not impossible but just more burdensome (as otherwise renegotiation would be meaningless), but they also seem to be based on the assumption that preservation of the contractual relationship is desirable, presumably on the basis of the argument that bringing the contract to an end would entail the burden of new transaction costs. This is, however, a questionable assumption to be carefully weighed against the benefits stemming from termination of the contract, which allows the parties to go back to the market in a competitive environment and seek anew, under the newly changed circumstances, for the most wealth-maximising transaction, either with the prior or with a new contractual counterparty.

Absent a contractual remedy, the attention of business actors turns to domestic options made available by PMRs.

### 3. The legal conundrum of domestic pandemic-mitigation restrictions

A common trait of PMRs is their main focus on domestic settings. With rare exceptions, the legislators’ attention seems to be directed to ensuring relief to the national economy, while international business transactions remain out of sight.^17^ Although the situation remains extremely fluid at the moment, PMRs generally contain either industry-specific provisions affecting contract non-performances (eg in the banking, tourism or housing sectors) or general provisions that apply to all outstanding contracts.^18^ Provisions of the latter kind are likely to be passed in an increasing number of countries.

Two of these provisions are noteworthy for their impact on the ordinary regime of *force majeure*.^19^ The one passed in Italy states that the respect of PMRs has to be “always” considered when assessing the exclusion of the debtor’s liability for failure to perform, with particular regard to deadlines and penal clauses.^20^ Remarkably, the norm is quite poorly drafted and lacks clarity, but nonetheless seems to defer to courts as to the determination of whether a specific PMR could excuse an obligor from performance, especially in respect of monetary obligations.^21^

Unlike Italy, the French legislator suspended all contractual clauses sanctioning a failure to perform from 12 March 2020 to one or two months (depending on the type of contract) after the revocation of the declaration of national emergency.^22^

Both provisions raise an important question about their general applicability to international business transactions, especially in the presence of appropriate *force majeure* clauses.

### 4. The impact of domestic measures on international contracts

The very different national PMRs pose puzzling conflict-of-laws questions, in that they all seem likely to be characterised by national governments as *overriding mandatory provisions* (*lois de police*), thus calling for their potential extraterritorial application in cross-border transactions.

Consider, for example, the Italian lockdown and the French suspension of all contract clauses sanctioning the failure to perform. On the one hand, an Italian company whose activity as a manufacturer of components is precluded by PMRs may certainly invoke *force majeure* against its Italian clients, but the situation could be less clear vis-à-vis foreign clients. Moreover, the Italian client that could not obtain the supply of components from the aforementioned manufacturer could be in an even more critical situation, likely to be treated differently based on whether its customers are Italian or foreign. On the other hand, the French suspension of all contract clauses sanctioning the failure to perform may be invoked against French counterparties, but may prove problematic vis-à-vis foreign parties.

The fact that the aforementioned PMRs are construed as “crucial” for a certain country’s “political, social or economic organisation”, as prescribed by the relevant European Union (EU) legislation in the definition of overriding mandatory provisions,^23^ is insufficient to ensure their extraterritorial effect, as the possibility of invoking such provisions is actually limited to the state of the forum and, to an even stricter extent, to the state of performance.^24^ Outside the EU, in most cases domestic courts would monitor the transaction only in light of their domestic public policy and mandatory laws, but would not take into account the overriding mandatory provisions (including PMRs) of other countries.

Therefore, in courts other than those of the state that enacted the PMRs, the measures would in most cases not be taken into consideration as overriding mandatory provisions, but could only operate as a relevant factual circumstance suitable to fulfil the requirements set in the relevant *force majeure* clause.^25^

Accordingly, the party whose business activity is directly affected by PMRs may invoke the *factum principis*, if the clause so allows, but the party that could not procure components due to the lockdown of its supplier will most likely be held liable for breach of contract on the grounds that it could have avoided the shortage of components by stocking them in advance or ensuring an alternative source from a country where no similar PMRs applied.

## III. Conclusion

This article attempted to test the suitability of existing *force majeure* clauses in international business transactions vis-à-vis the PMRs enacted in response to COVID-19. As they stand, PMR provisions are likely to add more complexity to the construction of *force majeure* clauses in a context that is already under enormous economic stress. The main source of this problem is that PMRs are passed at the national level and in an incremental way following the mounting emergency with absolutely no attention to the international dimension of COVID-19. As, regretfully, little has been done so far to propose harmonised legal solutions, the authors call for urgent and more coordinated efforts at the global level to tackle the impact of COVID-19 on international business transactions.

## Footnotes

1 “Business and the Pandemic: Covid Carnage”, *The Economist* (21 March 2020).

2

J Cohen and K Kupferschmidt , “Strategies Shift as Coronavirus Pandemic Looms” (2020) 367 Science 962 3210809310.1126/science.367.6481.962; A Wilder-Smith and DO Freedman , “Isolation, Quarantine, Social Distancing and Community Containment: Pivotal Role for Old-Style Public Health Measures in the Novel Coronavirus (2019-nCoV)” (2020) 27 Journal of Travel Medicine 1, 1 10.1093/jtm/taaa020PMC710756532052841.

3

D Heymann and N Shindo , “COVID-19: What Is Next for Public Health?” (2020) 395 The Lancet 542 10.1016/S0140-6736(20)30374-3PMC713801532061313, 543.

4

J Chen , “Pathogenicity and Transmissibility of 2019-nCoV – A Quick Overview and Comparison with Other Emerging Viruses” (2020) 22 Microbes and Infection 69 3203268210.1016/j.micinf.2020.01.004PMC7102641, 70; RM Anderson etal, “How Will Country-Based Mitigation Measures Influence the Course of the COVID-19 Epidemic?” (2020) 395 The Lancet 931 10.1016/S0140-6736(20)30567-5PMC715857232164834, 931.

5

C Huang etal, “Clinical Features of Patients Infected with 2019 Novel Coronavirus in Wuhan, China” (2020) 395 The Lancet 497 10.1016/S0140-6736(20)30183-5PMC715929931986264, 501.

6 Imperial College Covid-19 Response Team, “Impact of Non-Pharmaceutical Interventions (NPIs) to Reduce COVID-19 Mortality and Healthcare Demand” <https://www.imperial.ac.uk/mrc-global-infectious-disease-analysis/covid-19/report-12-global-impact-covid-19> (last accessed 2 April 2020) pp 14–16.

7 See A Spadaro’s contribution in this special issue.

8 Countries that, in Europe, have adopted the softer approach described in the text include, among others, The Netherlands, Sweden, Switzerland and the UK.

9

M Fontaine and F De Ly , Drafting International Contracts: An Analysis of Contract Clauses (Leiden, Martinus Nijhoff Publishers 2008) p 409 .

10

G Cordero-Moss , International Commercial Contracts: Applicable Sources and Enforceability (Cambridge, Cambridge University Press 2014 ) pp 109–16.

11 Fontaine and De Ly, supra, note 9, 453–92.

12

C Brunner , Force Majeure and Hardship under General Contract Principles: Exemption for Non-Performance in International Arbitration (Alphen aan den Rijn, Kluwer 2009) pp 383–89.

13
*Macromex Srl. v. Globex International Inc.* (AAA Final Award 23 October 2007) <http://cisgw3.law.pace.edu/cases/071023a5.html> (last accessed 2 April 2020).

14 English courts consider the requirement of “foreseeability” unnecessary, whereas French courts and other continental European courts consider it necessary; for a discussion under US law, see *TEC Olmos, LLC v. ConocoPhillips Co.* (Texas App. 31 May 2018), WL 2437449.

15 P Guiomard, “La grippe, les épidémies et la force majeure en dix arrêts”, *Dalloz Actualité* (4 March 2020) <https://www.dalloz-actualite.fr/flash/grippe-epidemies-et-force-majeure-en-dix-arrets#.XoX9qtMzZ-U> (last accessed 2 April 2020); *Kangwei Pharmaceutical v. Asia Pharmaceutical* (2012 Supreme People’s Court of the People’s Republic of China).

16 ICC Force Majeure and Hardship Clauses (25 March 2020) <https://iccwbo.org/publication/icc-force-majeure-and-hardship-clauses> (last accessed 2 April 2020).

17 The exception is Art 28(8) of *decreto-legge* 2 marzo 2020 n. 9 (*Misure urgenti di sostegno per famiglie, lavoratori e imprese connesse all’emergenza epidemiologica da COVID-19*).

18 Or neither, as is the case of countries that rely on the existing law, such as China. S Yu and X Liu, “China Issues Record Number of Force Majeure Certificates”, *Financial Times* (28 February 2020).

19 For Italy, see A Albanese, “Coronavirus e rapporti contrattuali in corso di esecuzione”, *Il Sole 24 Ore* (2 April 2020); for France, see J Heininch, “L’incidence de l’épidémie de coronavirus sur les contrats d’affaires: de la force majeure à l’imprévision” (2020) *Recueil Dalloz* 611, 612.

20 Art 91 of *decreto-legge* 17 marzo 2020 n. 18 (*Misure di potenziamento del Servizio sanitario nazionale e di sostegno economico per famiglie, lavoratori e imprese connesse all’emergenza epidemiologica da COVID-19*).

21 Heininch, supra, note 19, 613.

22 Art 4 Ordonnance no 2020-306 du 25 mars 2020 relative à la prorogation des délais échus pendant la période d’urgence sanitaire et à l’adaptation des procédures pendant cette même période.

23 Art 9 Regulation (EC) No 593/2008 of the European Parliament and of the Council of 17 June 2008 on the law applicable to contractual obligations (“Rome I”).

24 Cf. Art 9(2) and 9(3) Rome I Reg.

25 Case C135/15 *Hellenic Republic v. Grigorios Nikiforidis* (CJEU, 18 October 2016) [55].

